# Seroprevalence of anti-SARS-CoV-2 antibodies 6 months into the vaccination campaign in Geneva, Switzerland, 1 June to 7 July 2021

**DOI:** 10.2807/1560-7917.ES.2021.26.43.2100830

**Published:** 2021-10-28

**Authors:** Silvia Stringhini, María-Eugenia Zaballa, Nick Pullen, Javier Perez-Saez, Carlos de Mestral, Andrea Jutta Loizeau, Julien Lamour, Francesco Pennacchio, Ania Wisniak, Roxane Dumont, Hélène Baysson, Viviane Richard, Elsa Lorthe, Claire Semaani, Jean-François Balavoine, Didier Pittet, Nicolas Vuilleumier, François Chappuis, Omar Kherad, Andrew S. Azman, Klara Posfay-Barbe, Laurent Kaiser, Idris Guessous, on behalf of the Specchio-COVID19 study group

**Affiliations:** 1 Unit of Population Epidemiology, Division of Primary Care Medicine, Geneva University Hospitals, Geneva, Switzerland; 2 Department of Health and Community Medicine, Faculty of Medicine, University of Geneva, Geneva, Switzerland; 3 University Centre for General Medicine and Public Health, University of Lausanne, Lausanne, Switzerland; 4 Department of Epidemiology, Johns Hopkins Bloomberg School of Public Health, Baltimore, Maryland, United States; 5 Institute of Global Health, Faculty of Medicine, University of Geneva, Geneva, Switzerland; 6 Division of Laboratory Medicine, Geneva University Hospitals, Geneva, Switzerland; 7 Department of Medicine, Faculty of Medicine, University of Geneva, Geneva, Switzerland; 8 Infection Control Program and World Health Organization Collaborating Centre on Patient Safety, Geneva University Hospitals, Geneva, Switzerland; 9 Division and Department of Primary Care Medicine, Geneva University Hospitals, Geneva, Switzerland; 10 Division of Internal Medicine, Hôpital de la Tour, Geneva, Switzerland; 11 Department of Woman, Child, and Adolescent Medicine, Geneva University Hospitals, Geneva, Switzerland; 12 Geneva Centre for Emerging Viral Diseases and Laboratory Virology, Geneva University Hospitals, Geneva, Switzerland; 13 The members of this group are acknowledged at the end of the article

**Keywords:** Anti-SARS-CoV-2 antibodies, seroprevalence, population-based, Switzerland

## Abstract

**Background:**

Up-to-date seroprevalence estimates are critical to describe the SARS-CoV-2 immune landscape and to guide public health decisions.

**Aim:**

We estimate seroprevalence of anti-SARS-CoV-2 antibodies 15 months into the COVID-19 pandemic and 6 months into the vaccination campaign.

**Methods:**

We conducted a population-based cross-sectional serosurvey between 1 June and 7 July 2021, recruiting participants from age- and sex-stratified random samples of the general population. We tested participants for anti-SARS-CoV-2 antibodies targeting the spike (S) or nucleocapsid (N) proteins using the Roche Elecsys immunoassays. We estimated the anti-SARS-CoV-2 antibodies seroprevalence following vaccination and/or infection (anti-S antibodies), or infection only (anti-N antibodies).

**Results:**

Among 3,355 individuals (54.1% women; 20.8% aged < 18 years and 13.4% aged ≥ 65 years), 2,161 (64.4%) had anti-S antibodies and 906 (27.0%) had anti-N antibodies. The total seroprevalence was 66.1% (95% credible interval (CrI): 64.1–68.0). We estimated that 29.9% (95% Crl: 28.0–31.9) of the population developed antibodies after infection; the rest having developed antibodies via vaccination. Seroprevalence estimates differed markedly across age groups, being lowest among children aged 0–5 years (20.8%; 95% Crl: 15.5–26.7) and highest among older adults aged ≥ 75 years (93.1%; 95% Crl: 89.6–96.0). Seroprevalence of antibodies developed via infection and/or vaccination was higher among participants with higher educational level.

**Conclusion:**

Most of the population has developed anti-SARS-CoV-2 antibodies, despite most teenagers and children remaining vulnerable to infection. As the SARS-CoV-2 Delta variant spreads and vaccination rates stagnate, efforts are needed to address vaccine hesitancy, particularly among younger individuals and to minimise spread among children.

## Introduction

The Delta variant (Phylogenetic Assignment of Named Global Outbreak (Pango) lineage designation B.1.617.2) of the severe acute respiratory syndrome coronavirus 2 (SARS-CoV-2) drives a surge in new infections worldwide [1]. At the same time, vaccination rates stagnate in much of Europe [2], undermining efforts to achieve population immunity and curb the pandemic. Up-to-date seroprevalence estimates of anti-SARS-CoV-2 antibodies in the general population remain scarce, yet they are critical in monitoring the SARS-CoV-2 immune landscape in the population and guide public health decisions [3]. The state of Geneva, Switzerland, with a population of about 500,000, has been heavily affected by the pandemic, with 64,531 confirmed cases (127 per 1,000 inhabitants) and 748 deaths reported as at 27 August 2021 [4]. Previous serosurveys of the Geneva population revealed that one in 10 individuals had developed anti-SARS-CoV-2 antibodies by April–June 2020 following infection and two in 10 individuals had done so by November–December 2020 [5], before mass vaccination began.

**Figure 1:**
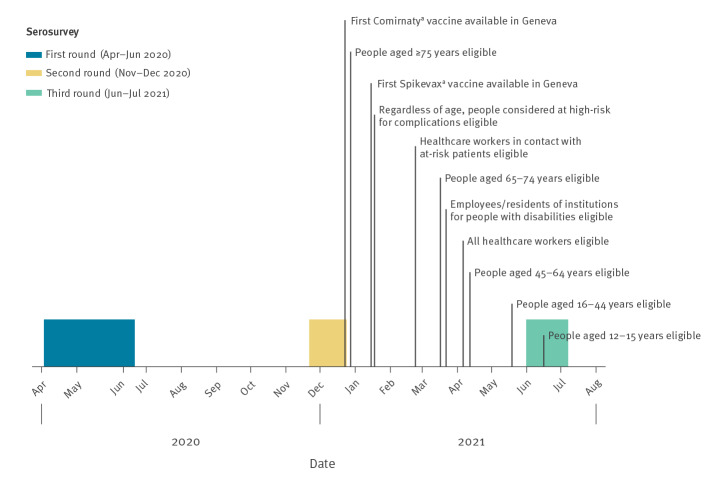
Timeframe of serosurveys and COVID-19 vaccination campaign in Geneva, Switzerland, April 2020–August 2021

The vaccination campaign began in Geneva on 28 December 2020. Initially only adults aged ≥ 75 years were eligible; in subsequent weeks, eligibility to receive the vaccine expanded to individuals with high risk of COVID-19-related complications, healthcare workers and progressively to younger age groups until reaching the 12–15-year-olds (Figure 1). During the course of this study, the only two vaccines approved for use in Switzerland were the Comirnaty (BNT162b2, mRNA, BioNTech-Pfizer, Mainz, Germany/New York City, United States (US)) and the Spikevax (mRNA-1273, Moderna, Cambridge, US).

COVID-19: coronavirus disease.

^a^ During the course of the three surveys (April 2020–July 2021), the two vaccines approved for use in Switzerland were Comirnaty (BNT162b2, mRNA BioNTech-Pfizer, Mainz, Germany/New York City, United States (US)) and Spikevax (mRNA-1273, Moderna, Cambridge, US) [14].

Eligibility to receive the vaccine allowed individuals to register for an appointment to receive the first vaccine dose.

To our best knowledge, the only serosurvey conducted after the third wave of the COVID-19 pandemic in a general population reported a total anti-SARS-CoV-2 antibodies seroprevalence of 17.3% in the Portuguese population up to March 2021 [6]. While they presented seroprevalence across three broad age categories, the study included no disaggregation of seroprevalence by sex or socioeconomic indicators, and its methodology did not allow distinguishing between antibodies developed following vaccination and/or infection and antibodies developed following infection only.

Using a representative sample of the general population, we aimed to assess the seroprevalence of anti-SARS-CoV-2 antibodies 15 months after the first confirmed case in Switzerland (26 February 2020) and 6 months after the vaccination campaign began.

## Methods

### Study design

We conducted a cross-sectional serosurvey between 1 June and 7 July 2021, recruiting participants from a random sample of individuals aged 0–64 years provided by the Swiss Federal Office of Statistics and an age- and sex-stratified random sample of individuals aged 18–24 years and ≥ 50 years from a previous serosurvey using the same methodology [5,7]. Newly selected individuals (aged 0–64 years) were invited by letter, while returning individuals (aged ≥ 18 years) were invited by letter or email when available. Individuals not having responded to the first invitation received up to two written reminders and were contacted by phone when available. Children and teenagers (aged < 18 years) were invited to participate alongside members of their household, while adults received individual invitations. Participation rates differed between age groups and depending on previous participation, ranging from 18.9% for newly-recruited children aged < 6 years (overall 23.7% participation rate among newly invited individuals) to 80.0% for returning participants aged ≥ 65 years (78.0% overall participation rate among returning participants) (Supplementary Figure S1). During the visit, participants provided a venous blood sample and completed a questionnaire that collected sociodemographic data, COVID-19-related medical history and vaccination information (Supplement S1).

### Immunoassays

To detect anti-SARS-CoV-2 antibodies, we used two commercially-available immunoassays: the Roche Elecsys anti-SARS-CoV-2 S immunoassay (Roche Diagnostics, Rotkreuz, Switzerland), which detects immunoglobulins (IgG/A/M) against the receptor binding domain of the virus spike (S) protein (#09 289 275 190, Roche-S), and has an in-house sensitivity of 99.6% (95% confidence interval (CI): 98.3–100) and specificity of 99.8% (95% CI: 99.3–100) [8,9]; and the Roche Elecsys anti-SARS-CoV-2 N immunoassay (Roche Diagnostics), which detects immunoglobulins (IgG/A/M) targeting the virus nucleocapsid (N) protein (#09 203 079 190, Roche-N), and has an in-house sensitivity of 99.8% (95% CI: 99.4–100) and specificity of 99.1% (95% CI: 98.3–99.7) [8]. We defined seropositivity using the manufacturer’s provided cut-off value of titer ≥ 0.8 U/mL for the Roche-S and cut-off index ≥ 1.0 for the Roche-N immunoassays.

### Statistical analyses

To estimate seroprevalence (% and 95% credible interval (Crl)), we expanded previous Bayesian modelling frameworks that accounted for age, sex, immunoassay performance and household clustering [5,7], jointly modelling the antibody response measured by the two immunoassays while additionally accounting for vaccination information. Since the vaccines used to date in Geneva elicit no response to the SARS-CoV-2 N protein [10], we used participants' two-marker antibody profiles to estimate the proportion having any anti-SARS-CoV-2 antibody and the proportion having antibodies because of infection (but could also have been vaccinated). Full details of the statistical model are provided in the Supplement S1.

To investigate potential socioeconomic disparities in seroprevalence, we estimated seroprevalence according to the highest obtained educational level among participants aged ≥ 18 years, and calculated the corresponding prevalence ratios and 95% CrI. We applied the probabilistic programming language Stan using the Rstan package and R version 4.1 (R Foundation, Vienna, Austria) [11].

### Ethical statement

The Geneva Cantonal Commission for Research Ethics approved this study (Project number 2020–00881). Informed written consent was obtained from all participants.

## Results

We included 3,355 participants, 2,497 first-time participants and 858 returning participants, of whom 54.1% were women, 20.8% were aged < 18 years and 13.4% were aged ≥ 65 years. Among adults, 8.1% had a primary education level and 59.5% had a tertiary education level (Table 1). Compared with the general population of Geneva, our sample had an overrepresentation of individuals with a tertiary education level (Supplementary Table S1).

**Table 1:** Sociodemographic characteristics of study participants, serological results and seroprevalence estimates, Geneva, Switzerland, 1 June–7 July 2021 (n = 3,355)

Characteristics		Participants		Vaccinated (self-reported)^a^		Seropositive^b^				Seroprevalence^c^			
						Anti-SARS-CoV-2 S protein		Anti-SARS-CoV-2 N protein		Antibodies of any origin		Antibodies of infectious origin	
		n	%	n	%	n	%	n	%	%	95% CrI	%	95% CrI
		3,355	100	1,449	43.2	2,161	64.4	906	27.0	66.1	64.1–68.0	29.9	28.0–31.9
Sex	Male	1,541	45.9	669	43.4	995	64.6	416	27.0	65.4	62.8–68.1	30.4	28.0–33.0
	Female	1,814	54.1	780	43.0	1,166	64.3	490	27.0	66.7	64.3–69.1	29.5	27.2–31.9
Age group (years)	0–5	150	4.5	0	0	32	21.3	29	19.3	20.8	15.5–26.7	20.8	15.5–26.7
	6–11	281	8.4	0	0	100	35.6	95	33.8	31.4	26.7–36.4	31.4	26.7–36.4
	12–17	266	7.9	5	1.9	99	37.2	91	34.2	41.0	35.6–46.4	37.7	32.5–43.1
	18–24	300	8.9	85	28.3	187	62.3	107	35.7	63.6	57.3–69.8	41.8	36.3–47.5
	25–34	372	11.1	121	32.5	212	57.0	97	26.1	61.1	56.1–65.9	31.9	27.8–36.2
	35–49	805	24.0	323	40.1	505	62.7	219	27.2	64.4	60.2–68.6	32.2	28.7–35.9
	50–64	732	21.8	517	70.6	609	83.2	196	26.8	84.7	81.1–88.1	29.8	26.5–33.4
	65–74	207	6.2	174	84.1	187	90.3	41	19.8	89.2	84.2–93.4	22.5	17.0–28.4
	≥ 75	242	7.2	224	92.6	230	95.0	31	12.8	93.1	89.6–96.0	16.2	11.8–21.1
Education level^d^	Primary	203	8.1	100	49.3	135	66.5	53	26.1	71.9	68.4–75.5	29.7	26.6–33.1
	Secondary	818	32.5	393	48.0	560	68.5	219	26.8	70.3	64.1–76.5	27.9	22.0–33.9
	Tertiary	1,499	59.5	878	58.6	1,134	75.7	381	25.4	75.2	72.6–77.9	31.7	28.9–34.4

Crl: credible interval; N: nucleocapsid protein; S: spike protein; SARS-CoV-2: severe acute respiratory syndrome coronavirus 2.

^a^ Self-reported having received at least one dose of the COVID-19 vaccine > 14 days before blood sample was drawn.

^b^ Serology based on Roche Elecsys anti-SARS-CoV-2 S immunoassay and N immunoassay (Roche Diagnostics, Rotkreuz, Switzerland), respectively.

^c^ Seroprevalence estimates reported as % and 95% credible interval, adjusted for test performance of both immunoassays and post-stratified to account for age distribution in the Geneva general population and for household clustering of infection and vaccination, n = 3,355 for total and sex- and age-stratified estimates; n = 2,520 for education-stratified estimates, excluding participants aged < 18 years. Seroprevalence of antibodies of any origin is based on proportion of participants with any anti-SARS-CoV-2 antibodies; seroprevalence of antibodies of infectious origin is based on proportion of participants who were naturally infected (but could also have been vaccinated).

^d^ Self-reported education level among participants aged ≥ 18 years (n = 2,520); missing education level values by age groups: (n = 15) in the group of 18–24-year-olds; (n = 49) in the group of 25–44-year-olds; (n = 53) in the group of 45–64-year-olds; (n = 2) in the group of 65–74-year-olds; and (n = 19) in the group of ≥ 75-year-olds.

Anti-SARS-CoV-2 antibodies were detected in all but three participants reporting having received at least one dose of the COVID-19 vaccine more than 14 days before serological assessment. Overall, 43.2% reported having received at least one COVID-19 vaccine dose > 14 days before their blood draw, 64.4% of all participants tested positive for anti-S antibodies, and 27.0% tested positive for anti-N antibodies (Table 1). The proportion of self-reported vaccinated participants was similar to that of the general population of Geneva across most age groups; study participants aged ≥ 70 years reported higher vaccination rates than their counterparts in the general population (Supplementary Table S2).

**Figure 2:**
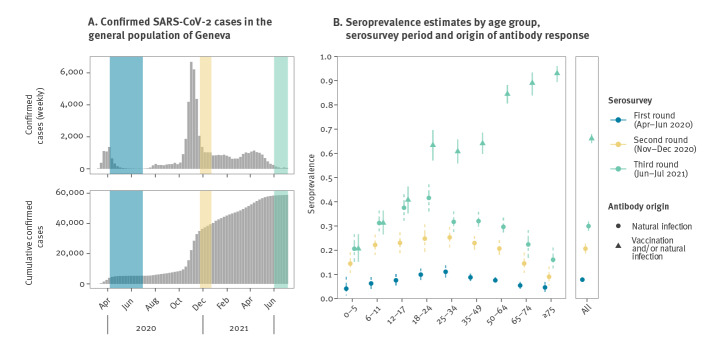
Confirmed SARS-CoV-2 infections and estimated seroprevalence of anti-SARS-CoV-2 antibodies, Geneva, Switzerland, March 2020–July 2021

The overall seroprevalence estimate was 66.1% (95% CrI: 64.1–68.0), including a seroprevalence of 29.9% (95% Crl: 28.0–31.9) for infection-derived antibodies (Figure 2). Estimates were similar across sexes, but varied widely across age groups, being lowest among children aged 0–5 years (20.8%; 95% Crl: 15.5–26.7) and highest among older adults aged ≥ 75 years (93.1%; 95% CrI: 89.6–96.0). In contrast, the seroprevalence of infection-induced antibodies was lowest among older adults aged ≥ 75 years (16.2%; 95% Crl: 11.8–21.1) and highest among young adults aged 18–24 years (41.8%; 95% Crl: 36.3–47.5) (Figure 2).

SARS-CoV-2: severe acute respiratory syndrome coronavirus 2.

A. Data presented in grey (available from: https://infocovid.smc.unige.ch) and serosurvey timings (coloured shaded). First serosurvey April–June 2020 (blue), in which the group of 0–5-year-olds only included children aged 5 years [7]; second serosurvey November–December 2020 (yellow) [5]; and third serosurvey June–July 2021 (green). B. Colour shades indicate timing of the serosurvey: first serosurvey April–June 2020 (blue), in which the group of 0–5-year-olds only included children aged 5 years [7]; second serosurvey November–December 2020 (yellow) [5] and third serosurvey June–July 2021 (green). Symbols indicate the antibody origin: dot indicates antibodies developed after infection (during the first two serosurveys, which took place before vaccination became available (see Figure 1), infection was the only possible antibody origin); triangle indicates antibodies developed after infection and/or vaccination. Vertical bars represent 95% credible intervals in seroprevalence estimations.

Among adults with tertiary education, 58.6% reported receiving at least one COVID-19 vaccine dose (Table 1 and Supplementary Table S3). In this group, 75.7% had anti-S antibodies, and 25.4% had anti-N antibodies. Among adults with up to a primary education, 49.3% reported receiving at least one COVID-19 vaccine dose, 66.5% had anti-S antibodies and 26.1% had anti-N antibodies. The overall seroprevalence was 75.2% (95% CrI: 72.6–77.9) among adult participants with tertiary education and 71.9% (95% Crl: 68.4–75.5) among those with up to a primary education (Table 1); the corresponding prevalence ratio for overall antibody seroprevalence was 0.85 (95% Crl: 0.77–0.93) among participants with up to primary education relative to those with a tertiary education level (Table 2).

**Table 2:** Prevalence ratio for seroprevalence of anti-SARS-CoV-2 antibodies, Geneva, Switzerland, 1 June–7 July 2021

Characteristics		Participants n	Antibodies of any origin			Antibodies of infectious origin		
			Prevalence ratio^a^	95% CrI^a^	p value	Prevalence ratio^a^	95% CrI^a^	p value
Sex	Male	1,541	1.01	0.94–1.08	0.75	1.01	0.92–1.11	0.81
	Female	1,814	1	NA		1	NA	
Age group (years)	0–5	150	0.34	0.25–0.44	< 0.0001	0.65	0.48–0.85	0.002
	6–11	281	0.52	0.43–0.61	< 0.0001	0.99	0.81–1.20	0.86
	12–17	266	0.67	0.57–0.78	< 0.0001	1.19	0.98–1.42	0.08
	18–24	300	1.04	0.92–1.17	0.49	1.32	1.10–1.57	0.003
	25–34	372	1	NA		1	NA	
	35–49	805	1.06	0.96–1.16	0.27	1.01	0.86–1.19	0.89
	50–64	732	1.39	1.28–1.52	< 0.0001	0.94	0.79–1.10	0.43
	65–74	207	1.47	1.34–1.60	< 0.0001	0.71	0.52–0.92	0.01
	≥ 75	242	1.53	1.41–1.67	< 0.0001	0.51	0.36–0.68	< 0.0001
Education level^b^	Primary	203	0.85	0.77–0.93	< 0.0001	0.93	0.81–1.07	0.28
	Secondary	818	0.84	0.72–0.96	0.01	0.88	0.69–1.08	0.22
	Tertiary	1,499	1	NA		1	NA	

Crl: credible interval; NA: not applicable; SARS-CoV-2: severe acute respiratory syndrome coronavirus 2.

^a^ Prevalence ratio (95% Crl) from Bayesian multinomial regression models accounting for sex, age, test performance and household clustering. Reference group for age and sex estimates (n = 3,355) is female, aged 25–34 years. For education level estimates (n = 2,520), reference group is female, aged 25–44 years with tertiary education level.

^b^ Self-reported education level among participants aged ≥ 18 years (n = 2,520).

## Discussion

In this seroprevalence survey we found that, by 7 July 2021, 66.1% of the population in Geneva had developed antibodies against SARS-CoV-2 after vaccination and/or infection. We also found that 29.9% of the population had antibodies following infection with SARS-CoV-2, three times more than the seroprevalence of 10.8% reported in April–June 2020 [7], and an 8.8% increase from the 21.1% seroprevalence reported in November–December, 2020 [5] (Supplementary Table S4). This increase in seroprevalence of infection-induced antibodies within a 6-month period was largest among young adults aged 18–24 years (16.1% increase) and teenagers aged 12–17 years (14.1% increase), indicating the third COVID-19 wave, comprised primarily of the Alpha (B.1.1.7) variant [12], may have particularly affected these age groups.

This is the first serosurvey providing seroprevalence estimates after the widespread availability of vaccination. We observed marked age differences in seroprevalence, which closely reflect the age-related infection risk observed in Geneva and elsewhere throughout the pandemic [5,7,13] and, subsequently, the progression of the age-dependent vaccination eligibility since December 2020 [14]. For instance, the 20.8% seroprevalence in children aged 0–5 years represents a 5.9% increase in a 6-month period which may indicate a lower infection risk compared to adults, and the fact that, to date, vaccination in Switzerland remains approved only for individuals aged ≥ 12 years. The 16.2% seroprevalence of infection-induced antibodies in adults aged ≥ 75 years mirrors their previously reported lower infection risk [5,7] and the fact that they were the first age group targeted by the vaccination campaign; with a reported 92.6% vaccination rate and an associated estimated total seroprevalence of 93.1%. It is also not possible to exclude that the lower proportion of participants with infection-induced antibodies in this age group is attributable to immunosenescence or higher likelihood of antibodies waning in the elderly population [15,16].

We also found that vaccination uptake differed by education level, with a higher proportion of individuals with tertiary education reporting being vaccinated than individuals with lower education levels, reflecting socioeconomic inequalities in vaccination as reported in the US and Israel [17,18]. The proportion having anti-S antibodies was correspondingly higher among individuals with tertiary education, though the proportion of anti-N antibodies was similar across education level groups, reflecting findings from a previous seroprevalence study [19], but differing from patterns of socioeconomic inequalities in infection risk observed in the United Kingdom, Germany and the US [20-22]. While a previous survey found no evidence of socioeconomic inequalities in infection-induced anti-SARS-CoV-2 antibody seroprevalence in the Geneva population [19], this study has indicated emerging inequalities in overall seroprevalence, likely driven by the higher vaccination uptake among the more socioeconomically privileged individuals. Indeed, several studies have found a clear pattern of socioeconomic disparities in vaccine hesitancy, whereby a lower proportion of socioeconomically disadvantaged individuals report intention or willingness to get vaccinated against COVID-19 than do more socioeconomically privileged individuals [23-28].

Strengths of this study include a large sample that is broadly representative of the general population in Geneva, the measurement of antibodies against both the SARS-CoV-2 S and N proteins and the robust novel modelling framework. Limitations include the fact that, as with most surveys [29], the sample population was generally more socioeconomically advantaged than the general population (Supplement S1), which may have led to overestimation of vaccine-derived antibody seroprevalence. However, the proportion of vaccinated individuals in our sample was similar to that observed in the general population of Geneva (Supplementary Table S2). Another limitation was that we only included formal residents and that we only assessed education as a socioeconomic indicator, which may have precluded the identification of inequalities based on other indicators. Finally, since we did not perform neutralisation assays, our estimates may not completely reflect protective immunity against SARS-CoV-2 [30].

## Conclusion

This study provides seroprevalence estimates of anti-SARS-CoV-2 antibodies in a broadly representative sample of the general population in Geneva after the third pandemic wave and the start of mass vaccination, distinguishing between antibodies developed following vaccination and/or infection, and antibodies developed following infection only. Our findings highlight how mass vaccination has closed the immunity gap in most of the adult population, particularly among older individuals who are at the greatest risk of severe COVID-19 outcomes. They attest to the effectiveness of free-of-charge vaccination programs in promoting immunisation against the virus while highlighting the need to strengthen efforts to address vaccine hesitancy. Importantly, our findings also show that the majority of children and teenagers, and a considerable proportion of young and middle-aged adults, lack anti-SARS-CoV-2 antibodies, leaving behind a large reservoir in the population to sustain transmission in the critical months to come. Finally, our findings indicate the emergence of socioeconomic inequalities in seroprevalence of anti-SARS-CoV-2 antibodies, likely driven by socioeconomically-related vaccine uptake.

## Supplementary Data

713000 Supplement
