# Supplementary material for: Seroprevalence of anti-SARS-CoV-2 antibodies 6 months into the vaccination campaign in Geneva, Switzerland, 1 June to 7 July 2021
Source: Euro Surveill. 2021 Oct 28;26(43):2100830. doi: 10.2807/1560-7917.ES.2021.26.43.2100830 (PMC8555371; doi:10.2807/1560-7917.ES.2021.26.43.2100830)
Supplement: Supplement [file 21-00830_STRINGHINI_Supplement.pdf]

Supplementary material

This supplementary material is hosted by *Eurosurveillance* as supporting information alongside the article *Seroprevalence of anti-SARS-CoV-2 antibodies 6 months into the vaccination campaign in Geneva*, *Switzerland, 1 June to 7 July 2021*, on behalf of the authors, who remain responsible for the accuracy and appropriateness of the content. The same standards for ethics, copyright, attributions and permissions as for the article apply. Supplements are not edited by *Eurosurveillance* and the journal is not responsible for the maintenance of any links or email addresses provided therein.

Supplementary information S1. Questions given to participants and used in present study, Specchio-COVID19 study

Question	Answer choices
What is your date of birth?	day / month / year
What is your sex?	Male / Female / Intersex
Vaccination	
Have you received the first dose of the vaccine against COVID-19?	Yes / No<br>If yes, date (day / month / year)
Have you received the second dose of the vaccine against COVID-19?	Yes / No<br>If yes, date (day / month / year)
Since the beginning of the pandemic, have you had a confirmed COVID-19 diagnosis, meaning having a positive COVID-19 from a nasal/throat swab, either RT-PCR or rapid antigenic test?	Yes / No<br>If yes, date (day / month / year
Educational level	
What is the highest level diploma/certification that you have obtained?	- None<br>- Primary school and/or orientation cycle<br>- Secondary education – *Maturité*/High School<br>- Professional training – Certified apprenticeship (*CFC*)<br>- Professional training – Non-certified apprenticeship<br>- Professional training – Higher professional degrees (post-*CFC*)<br>- Tertiary education – Bachelor/Master degree<br>- Tertiary education – Doctorate/PhD degree<br>- Other

S1. Overview of Statistical Framework

Our aim was to infer the proportion of the population having any antibody against SARS-CoV-2, as well as the proportion of those who acquired antibodies through natural infection as opposed to vaccination. We do so by modelling jointly the antibody response measured by the Roche-N and Roche-S immunoassays together with participants' responses to a vaccination questionnaire. We disentangle natural infection from vaccination antibody responses using the fact that the only available vaccines in Switzerland to date—the mRNA-1273 from Moderna/US NIAID,[5] and the mRNA-BNT162b2/Comirnaty from Pfizer/BioNTech[6]—both elicit a response exclusively to the S protein of SARS-CoV-2, as opposed to natural infections which typically elicit a response to both the N and S virus proteins. We expand previous Bayesian modelling frameworks used for seroprevalence estimates that account for demographic parameters (sex and age), test performance and household infection clustering.[1,2] The main additions to the previous models are that we now model jointly the response to both tests, and that we account for vaccination-induced antibody response.

S1.1 Multinomial response model

We model the Roche-S and Roche-N tests results for participant i, x_i, consisting of one of four possible outcome combinations $[n_{S^+N^+}, n_{S^-N^+}, n_{S^+N^-}, n_{S^-N^-}]$ (+ indicates antibody presence; − indicates absence) with $x_i \in \{[1,0,0,0],[0,1,0,0],[0,0,1,0],[0,0,0,1]\}$ using a multinomial distribution parametized by parameter vector $\pi_i = [\pi_i^{++}, \pi_i^{-+}, \pi_i^{+-}, \pi_i^{--}]$, where π^{lm} is the probability of having Roche-S test result l and Roche-N result m, accounting both for the underlying probability of each antibody status $p^{\pm/\pm}$, and test sensitivity, $\theta^+$and specificity, θ^-:

$$x_i \sim \mathrm{Multinomial}(\pi_i)$$

$$\pi_i^{++} = \theta_S^+\theta_N^+p_i^{++} + (1-\theta_S^-)\theta_N^+p_i^{-+} + \theta_S^+(1-\theta_N^-)p_i^{+-} + (1-\theta_S^-)(1-\theta_N^-)p_i^{--},$$

$$\pi_i^{-+} = (1-\theta_S^+)\theta_N^+p_i^{++} + \theta_S^-\theta_N^+p_i^{-+} + (1-\theta_S^+)(1-\theta_N^-)p_i^{+-} + \theta_S^-(1-\theta_N^-)p_i^{--},$$

$$\pi_i^{+-} = \theta_S^+(1-\theta_N^+)p_i^{++} + (1-\theta_S^-)(1-\theta_N^+)p_i^{-+} + \theta_S^+\theta_N^-p_i^{+-} + \theta_S^-(1-\theta_N^-)p_i^{--},$$

$$\pi_i^{--} = (1-\theta_S^+)(1-\theta_N^+)p_i^{++} + \theta_S^-(1-\theta_N^+)p_i^{-+} + (1-\theta_S^+)\theta_N^-p_i^{+-} + \theta_S^-\theta_N^-p_i^{--}.$$

The underlying probability of antibody status accounts both for the probability of natural infection λ_i and vaccination status, $v_i \in \{0,1\}$. Following previous modeling frameworks,[1,2] we model the probability of natural infection as a function of sex and age category, accounting for household infection clustering through a random effect, α_h:

$$logit(\lambda_i) = \alpha_h + \mathbf{X}_i\boldsymbol{\beta}$$

$$\alpha_h \sim Normal(0, \sigma_h^2)\ ,$$

where $\mathbf{X}_i$ is the matrix of covariates, and $\boldsymbol{\beta}$ the vector of regression coefficients. The probabilities of antibody status are then given by:

$$p_i^{++} = \gamma^{++}\lambda_i + \gamma^{-+}v_i$$

$$p_i^{-+} = \gamma^{-+}\lambda_i\ (1 - v_i)$$

$$p_i^{+-} = (1-\lambda_i)v_i + \gamma^{+-}\lambda_i$$

$$p_i^{--} = 1 - v_i(1-\lambda_i) - \lambda_i,$$

where $\gamma^{++}, \gamma^{-+}, \gamma^{+-}$ are the conditional probability of having S^+N^+, S^-N^+, S^+N^- responses, respectively, upon natural infection, v_i is the probability of having a vaccine-induced S^+ response as a function of the conditional probability of antibody response upon infection η_i, $v_i = \eta_i \times v_i$.

S1.2 Vaccination

To obtain population-level seroprevalence estimates, we also model the proportion of vaccinated individuals in each sex/age class following the approach used for natural infection:

$$v_i \sim \mathrm{Bernoulli}(\phi_i)$$

$logit(\phi_i) = \alpha_{v,h} + \mathbf{X}_i \boldsymbol{\beta_v}$

$\alpha_{v,h} \sim \text{Normal}(0, \sigma_v^2)$

Given vaccination policy recommendations in the state of Geneva, previously infected individuals were discouraged from being vaccinated in the early phase of the vaccination campaign, thus making the probability of vaccination dependent on the infection status of the individual. We account for this dependence by modelling separately the probability of vaccination given the infection status and marginalizing out the infection status:

$P(v_i|\boldsymbol{\Theta}) = \text{Bernoulli}(v_i|\phi_i^I) \curlywedge_i + \text{Bernoulli}(v_i|\phi_i^{\sim I})(1 - \curlywedge_i),$

$logit(\phi_i^{\sim I}) = \alpha_{v,h} + \mathbf{X}_i \boldsymbol{\beta_v},$

$logit(\phi_i^I) = \alpha_{v,h} + \mathbf{X}_i \boldsymbol{\beta_v} + \mathbf{X}_i \boldsymbol{\beta_v^I}$

$\alpha_{v,h} \sim \text{Normal}(0, \sigma_v^2),$

where $\boldsymbol{\Theta}$ is the vector of all model parameters, $I, \sim I$ indicates infection and non-infection, respectively, and $\boldsymbol{\beta_v^I}$ is the vector of regression coefficients giving the difference in probability of vaccination between infected and non-infected individuals.

When estimating the population-level seroprevalence, we account for the conditional probability of vaccination given non-infection, $p_{v|\sim}$, in the probability of a negative S and N response accounting for household vaccination clustering, p^{--}, as:

$p_{s,k}^{--} = 1 - p_{v|\sim I,s,k} \times (1 - p_{I,s,k}) - p_{I,s,k},$

where s, k denote the sex and age categories, $p_{v|\sim I,s,k} = \int_0^1 \emptyset_{s,k}^{\sim I}(t)dt = \int_0^1 \beta_{v,s,k} X_{s,k} + \sigma_v \Phi^{-1}(t)dt$, with $\Phi^{-1}(t)$ being the normal quantile function, and similarly, $p_{I,s,k}$ is the probability of infection with $p_{I,s,k} = \int_0^1 \curlywedge_{s,k}(t)dt = \int_0^1 \beta_{s,k} X_{s,k} + \sigma \Phi^{-1}(t)dt$.

S1.3 Diagnostic test performance

The individual performance of both N and S tests is incorporated hierarchically following Gelman & Carpenter[7]. The sensitivity, θ^+, is determined using n^+ RT-PCR positive controls from a laboratory validation study[8], of which x^+ tested positive. The specificity, θ^-, is determined using n^- pre-pandemic negative controls, of which x^- tested positive. For the Roche N test, these values are modulated by data in Ainsworth et al.[9]. For the Roche S test, the laboratory study data are modulated by those available on the Roche website (last accessed July 19, 2021).

S1.4 Priors

We follow a similar setting of the priors on the tests' sensitivity and specificity as Gelman & Carpenter[7]. For study j, the specificity θ_j^- and sensitivity θ_j^+ are drawn from normal distributions on the log odds scale:

$logit(\theta_j^-) \sim \text{Normal}(\mu_{\theta-}, \sigma_{\theta-})$

$logit(\theta_j^+) \sim \text{Normal}(\mu_{\theta+}, \sigma_{\theta+}).$

Hyperparameters μ_z and σ_z for $z \in (\theta^-, \theta^+)$ follow, on the logit scale, normal distributions $\mu_z \sim \mathcal{N}(4,2)$ and positive half-normals $\sigma_z \sim \mathcal{N}^+(0,1)$, respectively. These priors on test performance were identical for both the Roche S and Roche N tests.

We used standard normal $\mathcal{N}(0,1)$ priors for the logistic regression coefficients for infection $\boldsymbol{\beta}$. For coefficients of vaccination $\boldsymbol{\beta_v}$ and for coefficients of the difference in probability of vaccination between infected and non-infected individuals $\boldsymbol{\beta_v^I}$, we also used standard normal except for the youngest age group (ages 0-5 years and 6-11 years). For these two age groups, $\beta_v \sim \mathcal{N}(-10, 0.01)$ to reflect the fact that there was almost no vaccination in these youngest age groups in Geneva at the time of the study (NB vaccination registration for those aged 12-15 years opened on June 16, 2021:

https://www.ge.ch/en/getting-vaccinated-against-covid-19/covid-19-vaccination-campaign-geneva, last accessed July 20, 2021). Correspondingly, $\beta_v^I \sim \mathcal{N}(0, 0.01)$ for these two age groups.

The priors for the means of the household random effects α_h and $\alpha_{h,v}$, followed standard normal, and for standard deviations of the household random effects were positive half-normals, $\sigma_h \sim \mathcal{N}^+(0, 2)$ and $\sigma_v \sim \mathcal{N}^+(0, 2)$. We use a Dirichlet prior on the conditional probability of having S^+N^+, S^-N^+, S^+N^- responses upon natural infection, $\gamma^{++}, \gamma^{-+}, \gamma^{+-}$, $\gamma \sim \mathrm{Dir}(10, 1, 1)$, to highly favour production of both anti-S and anti-N antibodies upon infection. Finally, we put a strong prior on the conditional probability of antibody response after vaccination $\eta_i \sim \mathrm{Beta}(10, 0.1)$.

S1.5 Implementation

The model was coded in the probabilistic programming language Stan[10] using the Rstan package [11]. R [12] version 4.1 was used for data analysis. Four chains were run with 1500 iterations each, 250 of which were warmup, to give a total of 5000 posterior samples. Convergence was assessed by checking that $\widehat{R} \approx 1$, that the effective sample size was reasonable for all parameters, and visually using shinystan[13] diagnostics checks.

Supplementary Figure S1. Participants recruitment and inclusion into analytical sample

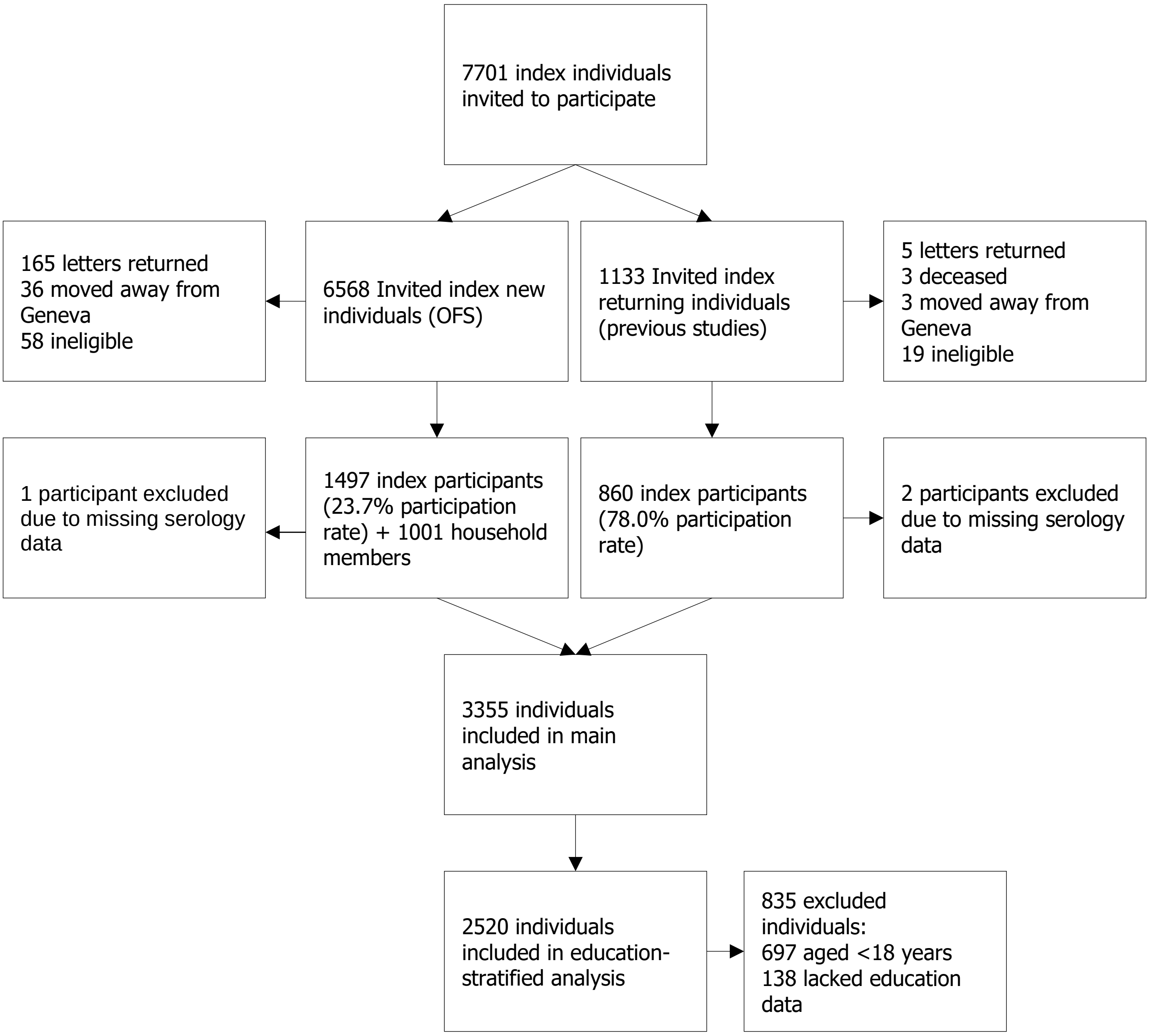

Supplementary Figure S2. Comparison of age and sex composition of study sample (bars) and the Geneva population (dots)

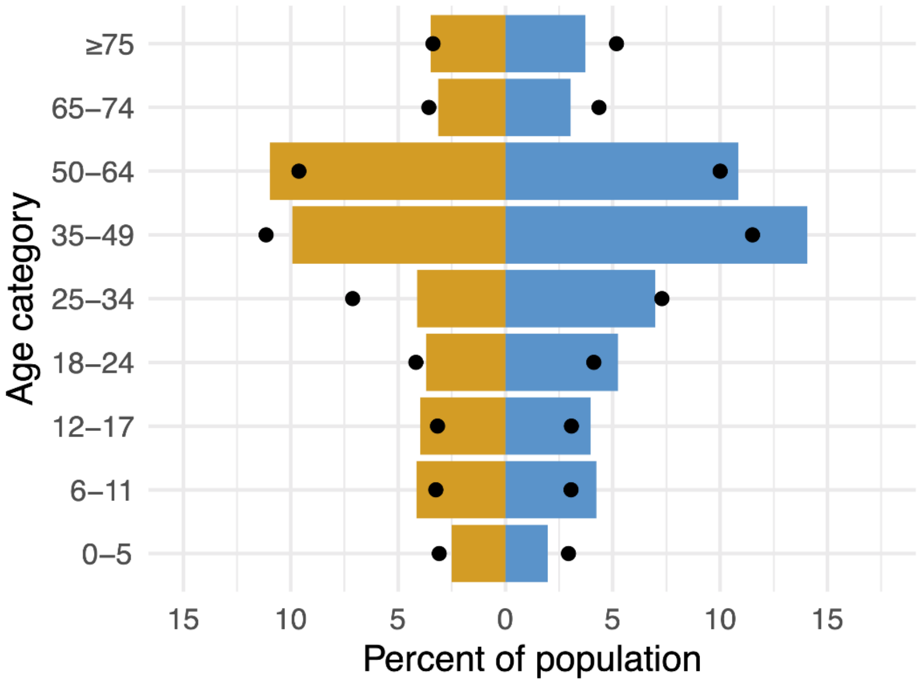

Dark yellow represents males; blue represents females.

Supplementary Figure S3. Quantitative values of Roche-S and Roche-N immunoassays results

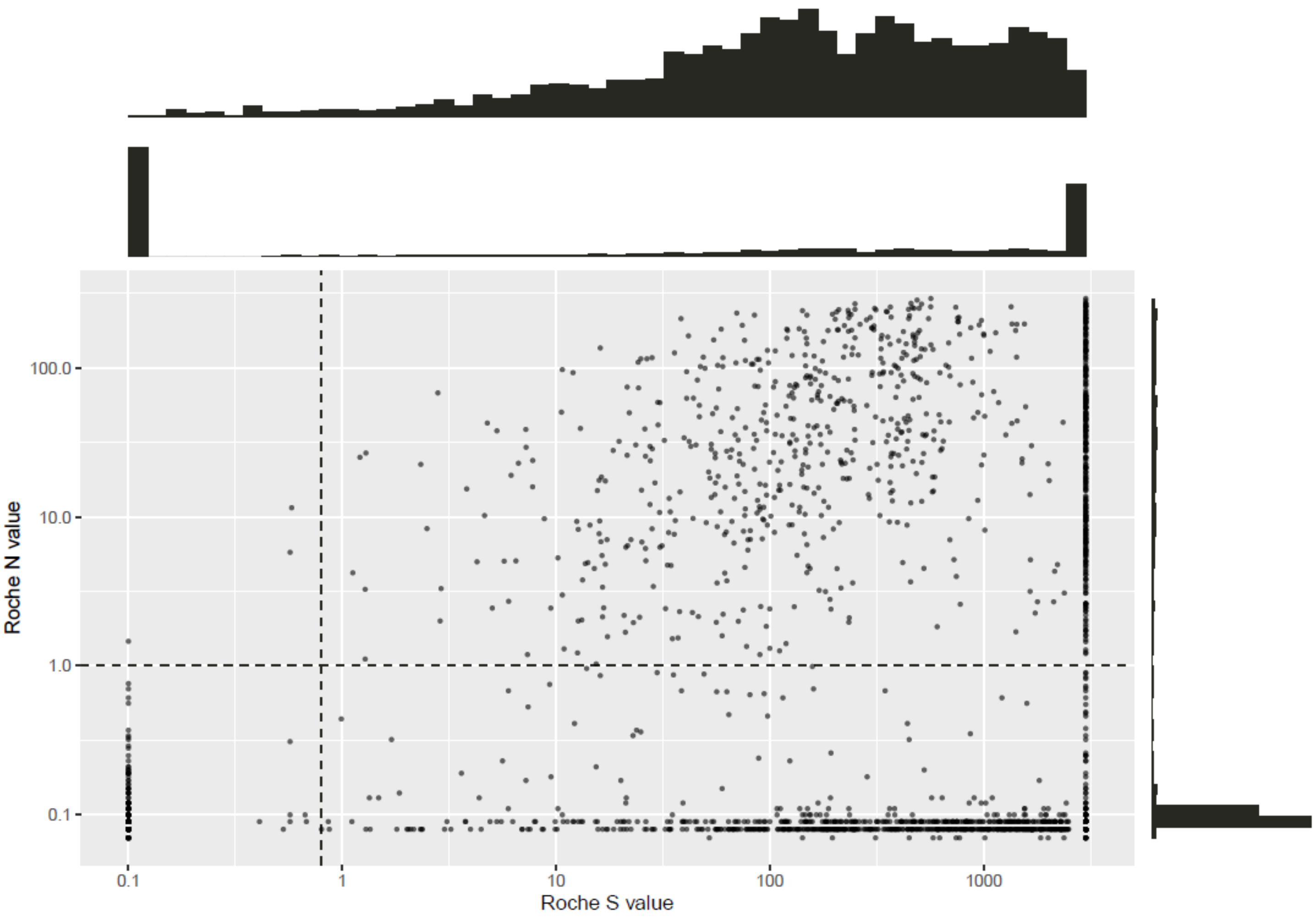

Each dot represents one participant. S value units are U/mL. For Roche-S, any values <0.4 were coded as 0.1, and any >2500 were coded as 3000 for ease of viewing, as our lab results do not provide more detailed data. The upper histogram for Roche S is thus a histogram without these 2 extremes i.e. binned from 0.4 to 2500 U/mL.

Supplementary Figure S4. Antibodies response category and vaccination status

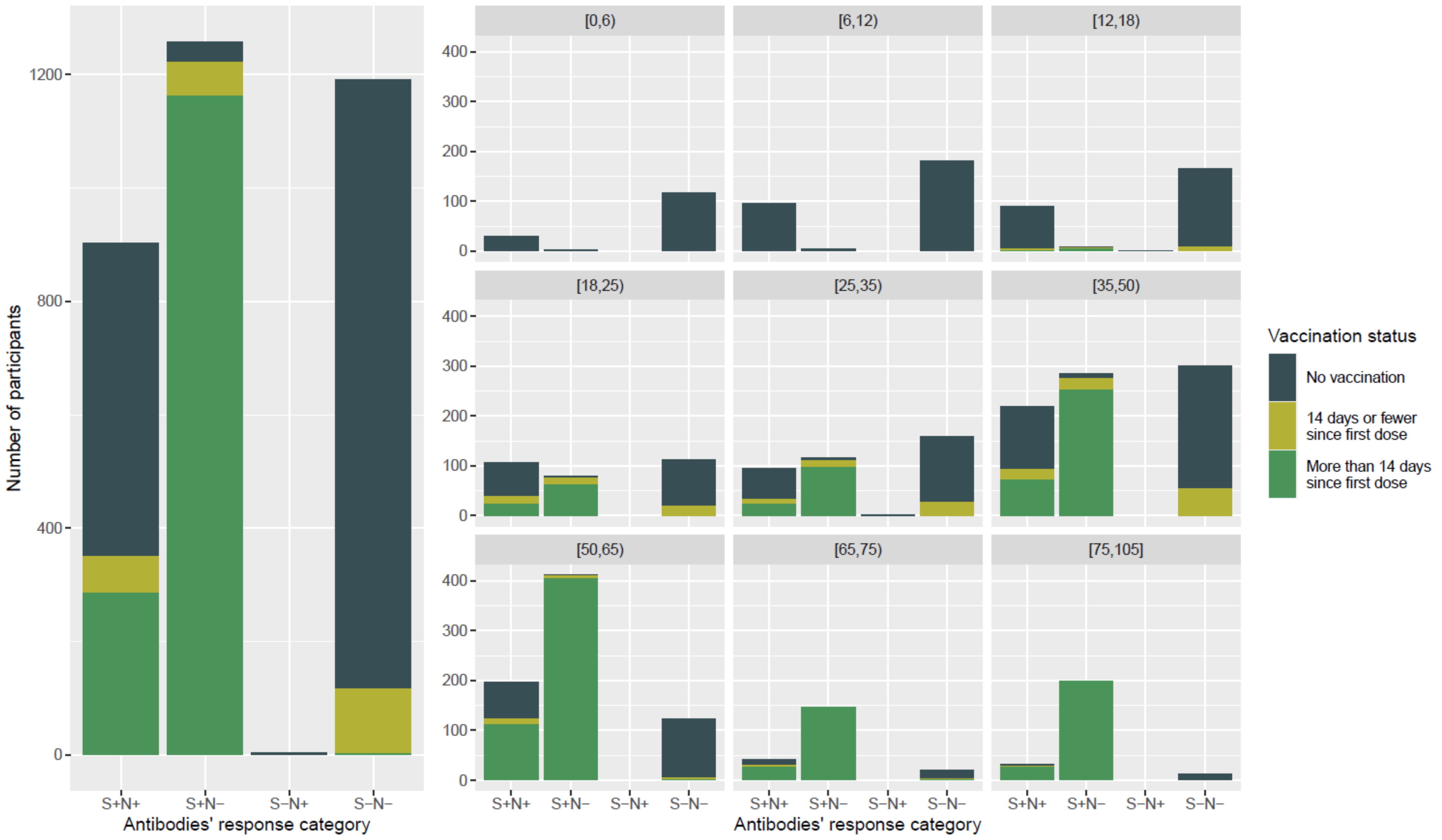

Number of participants in the four possible categories of the S and N tests. **+** indicates antibodies detected; **-** indicates antibodies not detected.

Supplementary Table S1. Comparison of education level in sample population and Geneva population

Education level	Geneva population No. (%)	Study sample No. (%)
Mandatory	98246 (26.6)	203 (8.1)
Secondary	118125 (32.0)	818 (32.5)
Tertiary	153334 (41.5)	1499 (59.5)

Geneva population data available from: https://www.ge.ch/statistique/domaines/15/15_03/tableaux.asp#1

Supplementary Table S2. Comparison of proportion vaccinated in sample population and Geneva population

	Study sample		Geneva population	
Age group, years	Individuals N	Vaccinated (self-reported) N (%)	Individuals N	Vaccinated N (%)
0-9	328	0 (0)	52912	13 (0.02)
10-19	444	56 (12.6)	53165	7604 (14.3)
20-29	306	131 (42.8)	65068	27492 (42.3)
30-39	423	189 (44.7)	76120	37679 (49.5)
40-49	558	312 (55.9)	76190	47030 (61.7)
50-59	505	363 (71.9)	71485	49217 (68.9)
60-69	304	241 (79.3)	46829	35281 (75.3)
70-79	236	218 (92.4)	36581	30053 (82.2)
≥80	81	75 (92.6)	25778	21338 (82.7)

Data on individuals vaccinated with at least 1 dose in study sample up to 4 July, 2021, to match data on individuals vaccinated in the general population of Geneva with at least 1 dose up to July 4, 2021.

Geneva population data available from:
https://www.covid19.admin.ch/en/vaccination/persons/d/demography?geo=GE&demoSum=total&demoAge=minOne

Supplementary Table S3. Proportion of participants having received at least 1 dose of the COVID-19 vaccine more than 14 days before serological assessment.

	Participants	Vaccinated, at least one dose	
	N (%)	Reported[a] N (%)	Estimated[b] % (95% CrI)
Total	3355	1449 (43.2)	44.9 (43.4-46.4)
Sex			
Male	1541	669 (43.4)	43.4 (41.3-45.5)
Female	1812	780 (43.0)	46.3 (44.5-48.1)
Age, y			
0-5	150	0 (0.0)	0.0 (0.0-0.0)
6-11	281	0 (0.0)	0.0 (0.0-0.0)
12-17	266	5 (1.9)	3.7 (1.9-6.2)
18-24	300	85 (28.3)	28.6 (23.8-33.6)
25-34	372	121 (32.5)	35.6 (31.1-40.2)
35-49	805	323 (40.1)	40.9 (37.3-44.5)
50-64	732	517 (70.6)	70.1 (66.7-73.3)
65-74	207	174 (84.1)	81.5 (75.8-86.5)
≥75	242	224 (92.6)	90.0 (86.0-93.5)
Education level[c]			
Primary	203	100 (49.3)	51.5 (48.5-54.4)
Secondary	818	393 (48.0)	52.9 (46.8-58.8)
Tertiary	1499	878 (58.6)	56.0 (53.6-58.4)

[a] Self-reported having received at least one dose of any COVID-19 vaccine, more than 14 days before blood drawing.

[b] Estimated vaccinated proportion in population, reported as % and 95% credible interval, adjusted for test performance of both immunoassays and post-stratified to account for age distribution in the Geneva general population and for household clustering of infection and vaccination.

[c] Self-reported education level among participants aged ≥18 years (N = 2520).

Supplementary Table S4. Comparison of seroprevalence of anti-SARS-CoV-2 antibodies[a] naturally developed through infection by November-December 2020 and June-July 2021, Geneva, Switzerland

	Geneva population N	Seroprevalence of antibodies after infection %		Percent increase	Absolute increase % points	Absolute increase N
		Nov-Dec 2020	Jun-Jul 2021			
Total	508774	21.1	29.9	42%	8.8	44772
Sex						
Male	246655	21.9	30.4	39%	8.5	20966
Female	262119	20.4	29.5	45%	9.1	23853
Age, y						
0-5	30633	14.9	20.8	40%	5.9	1807
6-11	32041	22.8	31.4	38%	8.6	2756
12-17	31726	23.6	37.7	60%	14.1	4473
18-24	42162	25.4	41.8	65%	16.4	6915
25-34	73285	25.9	31.9	23%	6.0	4397
35-49	115274	23.6	32.2	36%	8.6	9914
50-64	99841	21.2	29.8	41%	8.6	8586
65-74	40317	14.9	22.5	51%	7.6	3064
≥75	43495	9.3	16.2	74%	6.9	3001

[a] Seroprevalence based on results from Roche N test only.

Percent increase calculated as: ((Jun-Jul seroprevalence / Nov-Dec seroprevalence) – 1) x 100.

Absolute increase calculated as: Jun-Jul seroprevalence – Nov-Dec seroprevalence.

Absolute increase N calculated as: absolute increase % x Geneva population

Seroprevalence estimates for November-December 2020 from previous seroprevalence study [2].

Data on Geneva population available from: https://www.ge.ch/statistique/domaines/01/01_01/tableaux.asp#5

References

1. Stringhini S, Wisniak A, Piumatti G, Azman AS, Lauer SA, Baysson H, et al. Seroprevalence of anti-SARS-CoV-2 IgG antibodies in Geneva, Switzerland (SEROCoV-POP): a population-based study. The Lancet. 2020 Aug 1;396(10247):313–9.

2. Stringhini S, Zaballa M-E, Perez-Saez J, Pullen N, de Mestral C, Picazio A, et al. Seroprevalence of anti-SARS-CoV-2 antibodies after the second pandemic peak. The Lancet Infectious Diseases. 2021 mai;21(5):600–1.

3. Perez-Saez J, Zaballa M-E, Yerly S, Andrey DO, Meyer B, Eckerle I, et al. Persistence of anti-sars-cov-2 antibodies: immunoassay heterogeneity and implications for serosurveillance. Clinical Microbiology and Infection [Internet]. [cited 2021 Jul 21]; Available from: https://doi.org/10.1016/j.cmi.2021.06.040

4. L'Huillier AG, Meyer B, Andrey DO, Arm-Vernez I, Baggio S, Didierlaurent A, et al. Antibody persistence in the first 6 months following SARS-CoV-2 infection among hospital workers: a prospective longitudinal study. Clinical Microbiology and Infection. 2021 May 1;27(5):784.e1-784.e8.

5. Baden LR, El Sahly HM, Essink B, Kotloff K, Frey S, Novak R, et al. Efficacy and Safety of the mRNA-1273 SARS-CoV-2 Vaccine. N Engl J Med. 2020 Dec 30;384(5):403–16.

6. Polack FP, Thomas SJ, Kitchin N, Absalon J, Gurtman A, Lockhart S, et al. Safety and Efficacy of the BNT162b2 mRNA Covid-19 Vaccine. N Engl J Med. 2020 Dec 10;383(27):2603–15.

7. Gelman A, Carpenter B. Bayesian analysis of tests with unknown specificity and sensitivity. Journal of the Royal Statistical Society: Series C (Applied Statistics). 2020;69(5):1269–83.

8. Muench P, Jochum S, Wenderoth V, Ofenloch-Haehnle B, Hombach M, Strobl M, et al. Development and validation of the Elecsys Anti-SARS-CoV-2 immunoassay as a highly specific tool for determining past exposure to SARS-CoV-2. Journal of Clinical Microbiology. 2020;58(10):e01694-20.

9. Ainsworth M, Andersson M, Auckland K, Baillie JK, Barnes E, Beer S, et al. Performance characteristics of five immunoassays for SARS-CoV-2: a head-to-head benchmark comparison. The Lancet Infectious Diseases. 2020;20(12):1390–400.

10. Stan Development Team. Stan Modeling Language Users Guide and Reference Manual. Version 2.21.0. [Internet]. 2019. Available from: https://mc-stan.org

11. Stan Development Team. Rstan: the R interface to Stan. R package version 2.21.2 [Internet]. 2020. Available from: https://mc-stan.org

12. R Core Team. R: A Language and Environment for Statistical Computing. [Internet]. Vienna, Austria; 2021. Available from: https://www.R-project.org/

13. Gabry J. shinystan: Interactive Visual and Numerical Diagnostics and Posterior Analysis for Bayesian Models. R package version 2.5.0 [Internet]. 2018. Available from: https://CRAN.R- project.org/ package=shinystan
